# Attrition after Neoadjuvant Chemotherapy in Foregut Cancer: Experience at a Tertiary Center in the Deep South

**DOI:** 10.1245/s10434-025-17795-8

**Published:** 2025-07-17

**Authors:** Michelle Holland, Jaspinder Sanghera, Ioannis Liapis, Rida Ahmad, Krisha Amin, Ahmed Abdalla, Martin J. Heslin, Smita Bhatia, Annabelle L. Fonseca

**Affiliations:** 1 Department of Surgery, The University of Alabama at Birmingham, Birmingham, AL USA; 2 Department of Surgery, The University of South Alabama, Mobile, AL USA; 3 Institute for Cancer Outcomes and Survivorship, The University of Alabama at Birmingham, Birmingham, AL USA; 4 Department of Medical Oncology, The University of South Alabama, Mobile, AL USA

**Keywords:** Pancreatic cancer, Gastric cancer, Attrition, Deconditioning, Neoadjuvant chemotherapy

## Abstract

**Background:**

Neoadjuvant chemotherapy (NAC) is increasingly used in the management of foregut cancers to downstage tumors, treat micrometastases, and improve oncological outcomes. However, many patients fail to undergo surgical resection after NAC. This study aims to identify the underlying causes of non-tumor biology-related attrition and thus evaluate the potentially modifiable factors contributing to pre-surgical attrition.

**Methods:**

A retrospective review was conducted of patients with non-metastatic gastric or pancreatic adenocarcinoma treated between 2018–2022 at a tertiary and safety net hospital in the Southeastern U.S. Multivariable logistic regression and a root cause analysis (RCA) were performed to examine the association of sociodemographic factors with attrition and delineate underlying root causes.

**Results:**

Of 169 patients who received NAC, 47% (n = 80) experienced potentially modifiable attrition that was unrelated to disease progression. A diagnosis of pancreatic cancer (*p* = 0.001), age ≥ 75 (*p* = 0.04), and ≥ 3 ED visits after diagnosis (p=0.03) were independently associated with attrition on multivariable analysis. Four causes of non-tumor biology-related attrition were identified on RCA: physical deconditioning due to chemotherapy toxicity, malignancy or procedural complications, loss to follow-up resulting from missed appointments, healthcare delivery factors including delayed or absent referral to specialists, and patient refusal of treatment. Attrition was associated with significantly worse survival in both pancreatic and gastric cancer.

**Discussion:**

Nearly 50% of patients receiving NAC for pancreatic and gastric cancer failed to undergo surgery due to potentially modifiable causes. Addressing the underlying barriers through the implementation of structured prehabilitation programs, symptom management clinics, and cancer care navigators may reduce non-tumor biology-related attrition and improve outcomes.

Pancreatic and gastric cancers are estimated to be responsible for 5% of all new cancer cases and 10% of all cancer-related deaths, with 5 years survival rates as low as 13 and 36% respectively.^1,2^ Many patients present with metastatic or locally advanced disease, which preclude surgical resection.^3^ For nonmetastatic and potentially resectable cancer, neoadjuvant intent chemotherapy (NAC), defined as chemotherapy administered prior to curative intent surgery, is recommended in gastric cancer and being increasingly utilized in pancreatic cancer as well.^4,5^ The theoretical benefits of this approach include treating micro-metastasis, downsizing tumors, and improving patient selection for surgery prior to surgical resection with the goal of offering a survival advantage.^3,6–9^

Attrition after NAC poses a significant challenge, with 15–38% of patients failing to proceed to surgical resection after completion of NAC.^3,10–12^ Since NAC offers no survival advantage compared with up-front surgery without subsequent resection, addressing attrition for nontumor biology-related reasons presents a critical opportunity to enhance guideline-concordant treatment (GCT).^3^

This study aimed to identify and evaluate possible mechanisms leading to attrition, delineate root causes, and look for associations between sociodemographic and access-related factors. This essential first step to better understand the reasons for attrition after NAC will help with the future development of preventative strategies that will improve the delivery of care for patients with these cancers.

## Methods

### Study Population

Patients aged 18 years or older with a confirmed diagnosis of pancreatic or gastric cancer between January 2018 and December 2022, who received care at The University of South Alabama Health System, and had complete medical records, including demographic data, diagnostic imaging, treatment details, and follow-up outcomes, were included in this retrospective review. The University of South Alabama is a safety-net hospital and tertiary care facility providing care to a diverse population of more than 5 million people across southern and central Alabama, as well as the adjoining areas of the Gulf coast, which is home to a large rural and racial minority population.^13^ Patients were identified based on the International Classification of Disease (ICD) codes associated with the specific cancers: C25.0–C25.3, C25.7–C25.9 (pancreatic), and C16.0–C16.9 (gastric). The study was approved by the institutional review board at the University of South Alabama (IRB [1713166-2]).

### Data Collection

Data abstracted from the Electronic Health Record (EHR) included baseline patient characteristics, cancer-specific variables, and oncological treatment received were recorded. Cancer-specific variables included the type of cancer, relevant imaging information, clinical stage, and whether the workup was initiated by the patients’ primary care physician (PCP) or the emergency department (ED). Baseline patient characteristics included age, sex, race, insurance status, residence in rural or urban location, and presence of social support.

Area Deprivation Index (ADI), a validated neighborhood-level composite index based on 17 specific measures within the theoretical domains of income, education, employment, and housing quality captured in the American Community Survey, was used as an estimate of neighborhood disadvantage. The specific ADI score reflects the neighborhood deprivation relative to the remainder of the region of interest (state or nation).^14,15^ Given the relative poverty of Alabama compared with the nation, state ADI was used for this study.^16^ State ADI is a decile ranking at the block group level from 1 to 10 with higher scores indicating more deprivation. Scores were grouped into terciles: low (least deprivation), intermediate, and high (highest deprivation) ADI.^17^

Access-related variables included time from symptom onset to workup, time from diagnosis to first oncology appointment, and time from first oncology appointment to treatment initiation measured in weeks. Cancer treatment data points comprised the receipt of neoadjuvant and adjuvant chemotherapy, whether the patient underwent curative-intent surgical resection, reasons for nonreceipt of surgical resection, and number of and reasons for ED visits and hospitalizations after diagnosis, which were subsequently classified as either modifiable or nonmodifiable based on clinical review. Modifiable ED visits included those for malignancy-related pain, nausea/vomiting, dehydration, and symptomatic anemia, which were considered preventable with appropriate symptom management and chemotherapy side effect mitigation. Non-modifiable visits encompassed conditions, such as port site infections, deep vein thrombosis, procedure-related complications, pneumonia, gastrointestinal bleeding, altered mental status, COVID-19, chest pain, biliary, gastric outlet obstruction, and small bowel obstruction.

### Definition of Attrition and GCT

Attrition was defined as not receiving curative intent surgical resection after receipt of NAC for reasons other than tumor biology. Patients receiving NAC were stratified into two subcohorts: attrition and nonattrition. Progression on chemotherapy and locally advanced disease precluding surgery were excluded from further analysis, as this is likely driven by tumor biology and other nonmodifiable cancer-related characteristics. Patients who were documented to have comorbidities precluding resection prior to start of treatment were also excluded.

Guideline-concordant treatment (GCT) was defined based on National Comprehensive Cancer Network guidelines and varied based on type and stage of cancer, as has been described in a prior publication.^17^ In this patient cohort, GCT was defined as receipt of chemotherapy and curative-intent surgical resection, unless there was progression of disease on chemotherapy, or locally advanced disease precluding resection.

### Statistical Analysis

Simple descriptive statistics was performed on the overall data set comparing non-metastatic patients and patients that received NAC. Univariate analysis accompanied by *p*-values was performed to compare the two subcohorts of attrition and no attrition in the NAC group. *P* values were obtained by using Pearson’s chi-square test and Fischer’s exact test as appropriate for categorical variables, and Student’s *t*-test or Welch’s *t*-test as appropriate was utilized for the continuous variables. Kaplan-Meier survival curves were generated using available mortality data, stratified by cancer type and attrition status, and a log-rank test was used to determine statistical significance. Finally, a multivariable logistic regression model was developed to identify factors associated with attrition after NAC. Variable selection for the multivariable logistic regression model followed the purposeful selection method, beginning with univariate analyses (*p* < 0.2), and proceeding through iterative inclusion based on statistical significance (*p *< 0.1) and confounding effects. Multicollinearity was assessed using variance inflation factors (VIF); all VIFs were below 1.2, indicating minimal collinearity among the covariates of the model presented. Model fit was evaluated using the Hosmer-Lemeshow goodness-of-fit test.

### Root Cause Analysis

A root cause analysis (RCA) was performed to identify factors contributing to attrition utilizing a systematic approach previously described.^18,19^ In this study, an adverse event was defined as attrition after NAC. For patients meeting these criteria, an in-depth chart review was completed. Detailed information was collected from the patient’s medical records including onset of symptoms, workup, diagnosis, access to oncology expertise, overall treatment plan, treatment initiation and receipt, chemotherapy delays, ED presentations, hospitalizations, and missed oncological follow-up. If information on why the patient did not receive curative-intent surgery was explicitly available, this was recorded. Contributing factors were organized using the Ishikawa (fishbone) model by two authors (J.S. and M.H.), categorizing causes under major themes identified. Under each theme, specific causes or subcauses pertinent to the problem were detailed based on analysis of the collected data. Any disagreements regarding causal factors were resolved by the corresponding author, A.F. Solutions were then proposed to target each factor using supportive evidence from the literature.

## Results

**Fig. 1:**
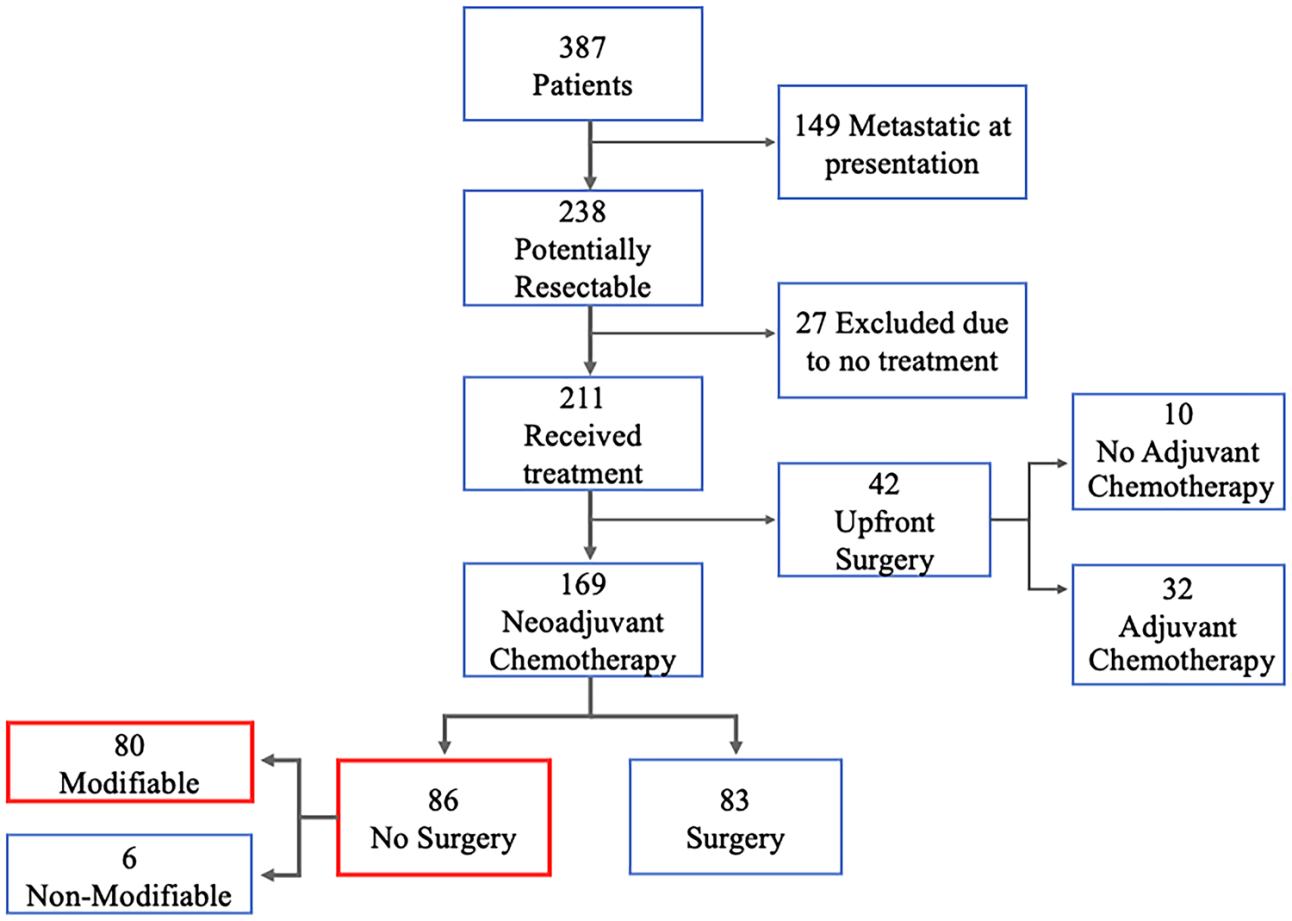
Attrition flow chart

A total of 387 patients with pancreatic and gastric cancer were seen at the institution during the study period, of which 238 (61%) patients had nonmetastatic disease that was deemed potentially resectable. Of these 238 patients, 167 (70%) had pancreatic adenocarcinoma and 71 (30%) had gastric adenocarcinoma with terminal outcomes depicted in Fig. 1; the majority were white (62%), male (55%), had Medicare insurance (58%), and had their workup initiated by a PCP (57%). Thirty-seven percent of patients lived in an area of low ADI, compared with 32% and 31% who lived in areas of medium and high ADI respectively. The median time from symptoms to the start of diagnostic workup was 6 weeks (interquartile range [IQR] 3–14), median time from diagnosis to first oncology appointment was 2.9 weeks (IQR 1.6–5.6), and median time from oncology appointment to treatment initiation was 3.1 weeks (IRQ 1.9–5.1). Patient sociodemographic and access-related variables are summarized in Table 1. There were no statistically significant differences noted between the overall cohort that was potentially upfront resectable and those that underwent NAC.

**Table 1:** Cohort characteristics

		Potentially resectable patients	Neoadjuvant intent chemotherapy
**Variable group**	**Characteristic**	**N = 238**^1^	**N = 169**^1^
**Sociodemographic**			
	**Age, yr,** n (%)		
	<55	36 (15%)	28 (17%)
	55–74	157 (66%)	108 (64%)
	≥75	45 (19%)	33 (20%)
	**Sex,** n (%)		
	Female	106 (45%)	69 (41%)
	Male	132 (55%)	100 (59%)
	**Race,** n (%)		
	White	148 (62%)	109 (64%)
	Black or African American	73 (31%)	48 (28%)
	Other	17 (7%)	12 (7%)
	**Insurance status,** n (%)		
	Private	59 (25%)	45 (27%)
	Medicare	137 (58%)	96 (57%)
	Medicaid	15 (6%)	10 (6%)
	Uninsured	17 (7%)	11 (7%)
	VA	10 (4%)	7 (4%)
	**Type of cancer,** n (%)		
	Gastric malignancy	71 (30%)	49 (29%)
	Pancreatic malignancy	167 (70%)	120 (71%)
	**Presence of PCP,** n (%)	140 (59%)	98 (58%)
	**Workup by,** n (%)		
	PCP	136 (57%)	97 (57%)
	ED	102 (43%)	72 (43%)
	**ED visits after presentation,** n (%)		
	<3 ED visits	206 (87%)	144 (85%)
	≥3 ED visits	32 (13%)	25 (15%)
	**Any social support,** n (%)	193 (82%)	142 (84%)
	**Distance (miles), m**edian (IQR)	27 (14, 64)	30 (16, 64)
	**From rural area,** n (%)	35 (15%)	24 (14%)
	**Deprivation (State ADI),** n (%)		
	Low	89 (37%)	64 (38%)
	Intermediate	76 (32%)	61 (36%)
	High	73 (31%)	44 (26%)
**Access related**			
	**Time from symptom onset to workup,** n (%)		
	<4 weeks	82 (34%)	61 (36%)
	4–6 weeks	33 (14%)	24 (14%)
	≥6 weeks	116 (49%)	79 (47%)
	Incidental	7 (3%)	5 (3%)
	**Time from diagnosis to first oncology appointment,** n (%)		
	<4 weeks	150 (63%)	116 (69%)
	4–6 weeks	34 (14%)	21 (12%)
	≥6 weeks	54 (23%)	32 (19%)
	**Time from first oncology appointment to treatment initiation,** n (%)		
	<4 weeks	128 (54%)	99 (59%)
	4–6 weeks	43 (18%)	37 (22%)
	≥6 weeks	44 (18%)	33 (20%)
	No Rx received	23 (10%)	0 (0%)
	**Time from symptom onset to treatment initiation,** n (%)		
	<4 weeks	8 (3%)	3 (2%)
	4–6 weeks	15 (6%)	13 (8%)
	≥6 weeks	192 (81%)	153 (90%)
	No Rx received	23 (10%)	0 (0%)

Of the 238 patients with potentially resectable disease, 211 (89%) received treatment and 27 (11%) received no treatment. For the patients that did not receive treatment 11 had no documented treatment plan, 13 were lost to follow-up before treatment initiation, two declined treatment, and reasons for lack of treatment was unknown for one patient. These patients were more likely to live in areas of high deprivation and have longer times from diagnosis to first oncology appointment.

Regarding the 211 patients that received treatment, 169 (71%) received NAC, whereas the remaining 42 (18%) received upfront surgery. Of the 169 patients who received NAC, 83 patients (49%) underwent curative intent resection, whereas 86 (51%) did not receive surgery, of which six (7%) did not receive surgery due to progression or locally advanced disease. The remaining 80 (47%) patients who did not receive surgery after NAC were deemed to have experienced modifiable attrition. Baseline demographic characteristics of this cohort, and those who underwent surgery or experienced modifiable attrition, are detailed in Table 2.

**Table 2:** Univariate analysis for attrition after NAC

		**After neoadjuvant therapy**		
**Variable group**	**Characteristic**	**No attrition** N = 83	**Attrition**^**∆**^ N = 80	***p***^a^
**Sociodemographic variables**				
	**Age, yr,** n (%)			0.153
	<55	18 (22%)	10 (12%)	
	55–74	52 (63%)	50 (63%)	
	≥75	13 (16%)	20 (25%)	
	**Sex,** n (%)			0.19
	Female	30 (36%)	37 (46%)	
	Male	53 (64%)	43 (54%)	
	**Race,** n (%)			0.372
	White	58 (70%)	48 (60%)	
	Black or African American	19 (23%)	26 (33%)	
	Other	6 (7%)	6 (7%)	
	**Insurance status,** n (%)			0.227
	Private	22 (27%)	22 (28%)	
	Medicare	43 (52%)	50 (63%)	
	Medicaid	5 (6%)	4 (5%)	
	Uninsured	7 (8%)	3 (4%)	
	VA	6 (7%)	1 (1%)	
	**Type of cancer,** n (%)			**0.002**
	Gastric malignancy	34 (41%)	15 (19%)	
	Pancreatic malignancy	49 (59%)	65 (81%)	
	**Charlson index, n (%)**			0.258
	2–3	20 (24%)	25 (32%)	
	≥4	62 (76%)	52 (68%)	
	**ECOG, n (%)**			**0.013**
	0	43 (52%)	24 (30%)	
	1	34 (41%)	44 (55%)	
	≥2	6 (7%)	12 (15%)	
	**Albumin (mg/dL), mean ± SD**	3.52 ± 0.66	3.35 ± 0.65	0.123
	**ED visits, n (%)**			**0.021**
	<3 ED visits	76 (92%)	63 (79%)	
	≥3 ED visits	7 (8%)	17 (21%)	
	**Workup by,** n (%)			0.56
	PCP	45 (54%)	47 (60%)	
	ED	38 (46%)	33 (40%)	
	**Any social support,** n (%)	71 (86%)	66 (83%)	0.596
	**Distance (miles),** mean ± SD	53 ± 56	48 ± 49	0.524
	**Distance (hours),** mean ± SD	1.06 ± 0.96	0.89 ± 0.74	0.21
	**From rural area,** n (%)	10 (12%)	12 (15%)	0.581
	**Deprivation (State ADI),** n (%)			0.078
	Low	38 (46%)	23 (29%)	
	Intermediate	25 (30%)	33 (41%)	
	High	20 (24%)	24 (30%)	
**Access to care variables**				
	**Time from symptom onset to workup (weeks),** Mean ± SD	8 ± 8	11 ± 14	0.15
	**Time from diagnosis to first oncology appointment (weeks),** mean ± SD	3.5 ± 3.6	4.5 ± 5.7	0.195
	**Oncology visit to treatment (weeks),** mean ± SD	4.9 ± 6.6	4.4 ± 3.1	0.518
	**Time from symptom onset to treatment initiation (weeks),** mean ± SD	18 ± 13	23 ± 22	0.092

^a^Pearson’s chi-squared test; two-sample *t*-test; Fisher’s exact test; Welch two-sample *t*-test

∆ After exclusion of patients who progressed on NAC

*SD* standard deviation

On univariate analysis, the attrition cohort had significantly more pancreatic cancer patients (*p *< 0.002). Though not statistically significant, there was a larger portion of patients aged 75 or older in the attrition subcohort (25% vs. 16%). Furthermore, the majority of patients in the non-attrition subcohort lived in an area of low ADI (46%), whereas most patients in the attrition subcohort lived in an area of intermediate ADI (41%), although this was also not statistically significant. In the analysis of access-to-care variables, patients who experienced attrition demonstrated longer time intervals across measured domains compared to those without attrition, although none reached statistical significance. Specifically, the mean time from symptom onset to initial workup was 11 weeks in the attrition group versus 8 weeks in the non-attrition group (*p *= 0.15). Time from diagnosis to first oncology visit was also longer in the attrition group (4.5 vs. 3.5 weeks, *p *= 0.195), whereas time from oncology visit to treatment initiation was slightly shorter (4.4 vs. 4.9 weeks, *p *= 0.518). The overall interval from symptom onset to treatment initiation was notably longer in the attrition group (23 vs. 18 weeks, *p *= 0.092). Furthermore, regarding modifiable ED visits, 59% of ED visits in the attrition were modifiable/intervenable compared with 44.6% of ED visits in the non-attrition cohort.

**Fig. 2:**
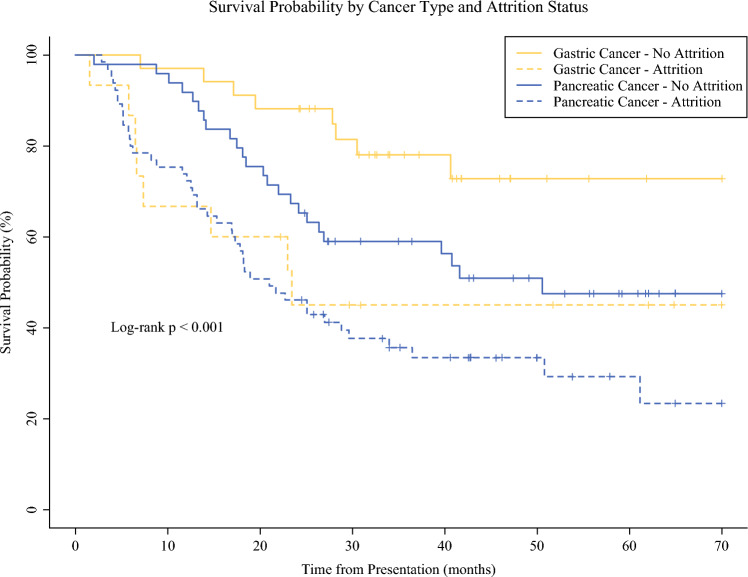
Survival probability by cancer type and attrition status

Figure 2 depicts Kaplan-Meier survival curves stratified by cancer type and attrition status among patients who received neoadjuvant chemotherapy. Patients with gastric cancer who did not experience attrition demonstrated the most favorable survival, followed by pancreatic cancer patients without attrition. In contrast, patients who experienced attrition, regardless of cancer type, had the lowest overall survival. A log-rank test confirmed that the differences in survival among the four groups were statistically significant (*p *< 0.001).

Table 3 depicts the multivariable model for factors associated with attrition post-NAC. Pancreatic cancer (odds ratio [OR] 3.83, 95% confidence interval [CI] 1.76–8.81), age at diagnosis ≥ 75 years (OR 3.3, 95% CI 1.08–10.7), and three or more ED visits after diagnosis (OR 3.25, 95% CI 1.16–10) were independently associated with non-tumor biology-related attrition. Race and state ADI were not statistically significantly associated with attrition.

**Table 3:** Multivariate analysis for attrition after NAC

Characteristic	OR	95% CI	*p*
**Race**			
White or other	–	–	
Black	1.54	0.67, 3.61	0.313
**Type of cancer**			
Gastric malignancy	–	–	
Pancreatic malignancy	3.83	1.76, 8.81	**0.001**
**Age, yr**			
<55	–	–	
55–74	1.63	0.64, 4.31	0.309
≥75	3.30	1.08, 10.7	**0.04**
**Deprivation**			
Low	–	–	
Intermediate	2.23	1.00, 5.13	0.054
High	1.90	0.71, 5.19	0.202
**ED visits after presentation**			
<3 ED visits	–	–	
≥3 ED visits	3.25	1.16, 10.0	**0.03**

*OR* odds ratio, *CI* confidence interval, *ED* emergency department

### Root Cause Analysis

**Fig. 3:**
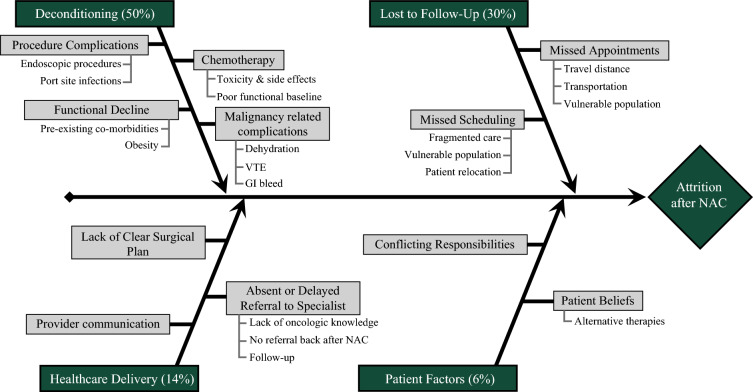
Root cause analysis

The Fishbone model demonstrating root causes of modifiable attrition is shown in Fig. 3. Underlying causes of attrition can be overarchingly categorized into deconditioning, loss to follow-up, healthcare delivery, and patient factors.

The most prevalent cause of attrition was physical deconditioning (50%). Primary contributors were chemotherapy-related toxicity (38%), procedural complications from cancer-specific procedures such as post-ERCP pancreatitis and port-related complications (4%), malignancy-related complications including dehydration, gastrointestinal bleeds and thromboembolic events that resulted in hospitalization (7%), and functional decline due to exacerbation of preexisting comorbidities (1%).

Loss to follow-up, which was the second most prevalent cause of attrition at 30%, was noted to be due to either missed appointments (19%) or lack of scheduled follow-up (11%). Patients in the former group had multiple (2 or more) missed appointments, and long travel distances and/or lack of social support, and there was evidence of health system attempts to reestablish contact or reschedule appointments. Patients in the latter group were initially seen by a surgical oncologist and started on NAC; however, they did not have a follow-up appointment scheduled at the initial visit, and no record of the healthcare system attempting to reestablish contact was found.

Healthcare delivery factors were the underlying cause of modifiable attrition in 14% of patients. Thus, 6% experienced delayed or non-referral to surgical oncology, often due to inadequate knowledge (e.g., misdiagnosing liver hemangiomas as metastatic cancer, and failing to refer patients with resectable disease for surgical evaluation). An additional 8% were evaluated by a surgical oncologist, but there was no documented discussion or plan for surgical resection at this visit and no subsequent visit was scheduled to reassess for resectability after NAC. In all these patients, reasons for this could not be ascertained from the chart or be ascribed to anatomic unresectability or patient-related comorbidities precluding resection.

Patient factors (6%) included belief in alternative therapies in 2% of the patients and conflicting responsibilities in 4%, where patients declined surgery owing to inadequate social support or the need to prioritize other obligations, such as caring for a chronically ill spouse, which they saw as precluding themselves from the recovery time needed for an operation.

## Discussion

This retrospective study sheds light on the multifactorial barriers contributing to attrition after NAC in patients with pancreatic and gastric cancer. Nearly half the patients in this study who initiated NAC failed to undergo curative-intent surgical resection, largely due to modifiable factors, emphasizing the critical need for targeted interventions to optimize treatment adherence and outcomes. While attrition due to tumor progression is well-documented and may indeed be a reason to avoid an unnecessary operation, the high rate of nontumor biology-related modifiable attrition seen in this study highlights the opportunity to address patient-, provider-, and system-level barriers.

This study observed a lower rate of tumor progression-related attrition compared to published literature, where progression accounts for 25–30% of attrition.^10^ This discrepancy may reflect the fact that the study excluded patients with documented comorbidities precluding resection prior to the start of treatment, and the detailed root cause analysis performed identified more granular modifiable factors contributing to significant delays in care prior to disease progression. Additionally, the study site is a safety-net hospital in southern United States, serving a population with significant socioeconomic challenges and poorer health outcomes,^20,21^ likely amplifying the role of sociodemographic factors contributing to attrition.

The association between pancreatic cancer and higher attrition rates underscores the aggressive nature of pancreatic malignancies and the physical toll imposed by neoadjuvant therapies. Patients with pancreatic cancer are at risk for chemotherapy-related toxicity and disease-related complications, contributing to functional decline and subsequent ineligibility for surgical resection. Additionally, the complexity of pancreatic surgery, particularly when vascular involvement is present, necessitates a higher preoperative functional status, further contributing to the observed attrition rates.^22^ These findings underscore the importance of proactive measures, such as structured prehabilitation programs, to optimize patients’ functional status before and during NAC.

Increased ED utilization may serve as a surrogate for patients’ lack of access to coordinated outpatient cancer care and primary care despite ongoing cancer treatment. Primary care providers (PCP) may help coordinate cancer care, manage treatment-related side effects, optimize comorbid conditions, and deliver psychosocial support, thus acting as a safety-net for patients, with studies reporting that PCPs may have greater contact with patients than their oncology providers during treatment.^23–25^ Poor care coordination and inadequate information sharing between providers or across systems has been linked to worse outcomes.^26–28^ Enhancing the integration of PCPs within oncology care pathways and improving communication between primary and secondary care may provide better continuity of care and reduce ED utilization while decreasing overall cost of care.

Regarding survival differences, the gradient in survival identified in this study is consistent with the known clinical behavior of each malignancy, and likely reflects the inherent differences in tumor biology and treatment-related burden. Pancreatic cancer is widely recognized as having a more aggressive clinical course, with faster progression, lower resectability rates, and limited therapeutic responsiveness, which collectively contribute to its poorer prognosis.^1,2,36,37^ While the neoadjuvant regimens in both malignancies (in particular FOLFIRINOX for pancreatic cancer or FLOT for gastric cancer) are associated with significant toxicity, the surgical burden differs markedly, which adds an additional layer of complexity in the management of pancreatic cancer. These findings highlight the critical need to optimize supportive care and symptom management during neoadjuvant therapy, while also underscoring the importance of gaining a deeper understanding of the underlying factors that contribute to treatment attrition.

Root cause analysis demonstrated deconditioning to be the most common cause of attrition. Deconditioning was found to be due to chemotherapy-related toxicity, and procedural or malignancy-related complications. Structured prehabilitation programs incorporating physical therapy and nutritional support can maintain or improve functional reserves and are another strategy to decrease deconditioning.^11,29^ Investment in infusion and symptom management clinics will allow for prompt treatment of malignancy-related pain or dehydration, thus reducing unnecessary hospitalizations, treatment delays and subsequent deconditioning related attrition.^30,31^

Loss to follow-up emerged as another significant modifiable factor contributing to attrition, where patients had initial contact with specialist oncological services, but did not have a follow-up appointment scheduled, or missed a follow-up appointment without any further health system attempt to re-establish contact. Patients with transportation barriers and inadequate social support were more likely to miss follow-up appointments, highlighting the impact of logistical and socioeconomic barriers on treatment adherence. Implementing automated reminders and leveraging telehealth services can help address these barriers and reduce the travel burden. Cancer care navigators can serve as a pivotal link to enhance continuity and provide support for patients navigating complex treatment schedules. This is crucial particularly among patients with lower health literacy, who are more likely to forego or delay care due to financial costs, life responsibilities, transportation issues, and fear around diagnosis.^32^ Cancer care navigators can be instrumental in connecting patients with support services and resources to mitigate these barriers.^33^ Furthermore, they can actively arrange follow-up appointments, ensure there is a clear surgical plan, promote interdisciplinary communication, ameliorate scheduling issues, address any financial or transportation barriers that patients face, and contact patients who miss appointments. Indeed, Kronefield et al. noted that a cancer care navigator was an independent predictor of improved survival in gastric adenocarcinoma.^11^

Healthcare delivery factors included delayed referral or non-referral to surgical oncology that appeared to be driven by inadequate provider knowledge concerning resectability, underscoring the need for standardized referral protocols and multidisciplinary tumor boards to ensure timely surgical evaluations for all eligible patients. Furthermore, improving communication between medical oncologists and surgical oncologists is essential to prevent gaps in care that may arise when patients transition between different specialties. This is especially important for community oncologists who may not have easy access to tumor boards or surgical oncologists within their facilities. Collaboration with physicians outside the health system is critically important, as are educational initiatives designed to enhance providers’ knowledge of resectability criteria and the importance of timely surgical referral.

Patient refusal of surgery, although less common, stemmed from patients seeking alternative treatments or having competing responsibilities that precluded them from receiving surgical resection. It is estimated that patient refusal accounts for 1–12% of presurgical attrition in patients with resectable foregut cancer and is associated with other sociodemographic and access-related factors such as Black race, older age, and lack of insurance.^10,34,35^ Poor health literacy, cultural beliefs, inadequate social support, and viewing surgery as futile may help explain these observed differences.^35^ Addressing patient refusal requires a nuanced approach that considers individual preferences and cultural beliefs. Shared decision-making tools that provide patients with comprehensive, culturally sensitive information about the risks and benefits of surgery can empower patients to make informed choices. Additionally, offering social support services, such as transportation assistance and caregiver support programs, may help alleviate some of the practical barriers that may influence patients’ decisions to decline surgery. In addition to personal beliefs and competing responsibilities, refusal of surgery may also be attributable to other factors, such as medical mistrust and patient-provider communication.

This study should be interpreted in light of several limitations. As a retrospective analysis, it is constrained by the data available in the EHR, particularly for patients who received part of their treatment at outside facilities, where records were not readily accessible. Additionally, being conducted at a single tertiary care center in the southeastern United States, its generalizability to other regions or healthcare settings may be limited and future studies should examine attrition rates of other institutions and geographical areas within the United States. However, the root causes of modifiable attrition during NAC likely exist in other safety-net hospitals, suggesting that the proposed solutions may have broader applicability.

Future research should explore patients’ perspectives and patient-reported barriers to accessing surgery after NAC. A mixed-methods approach, combining qualitative insights and quantitative data, could help to develop predictive models to identify patients at risk for attrition early and ensure they receive appropriate support.

## Conclusions

This study provides valuable insight into the underlying modifiable causes of attrition after NAC in patients with pancreatic and gastric cancer. Non-tumor biology related attrition was largely driven by deconditioning, patient loss to follow-up, healthcare delivery, and refusal of surgery. Patient age, increased ED utilization, and pancreatic malignancies were predictive of attrition. Identifying at-risk patient populations, improving provider communication, implementation of structured prehabilitation programs, cancer care navigation services, and automated health system alerts could help decrease attrition during NAC.
